# Aggressive Behaviors in Alzheimer Disease and Mild Cognitive Impairment: Systematic Review and Meta-Analysis

**DOI:** 10.1016/j.jagp.2018.10.008

**Published:** 2019-03

**Authors:** Rongqin Yu, Anya Topiwala, Robin Jacoby, Seena Fazel

**Affiliations:** Department of Psychiatry (RY, AT, RJ, SF), Warneford Hospital, University of Oxford, Oxford, England

**Keywords:** Alzheimer disease, mild cognitive impairment, aggressive behaviors, systematic review

## Abstract

**Objective:**

We aim to estimate the risk of perpetrating aggression in Alzheimer disease (AD) and mild cognitive impairment (MCI) by conducting a systematic review and meta-analysis of primary studies.

**Methods:**

A systematic search was conducted in six bibliographic databases according to a preregistered protocol. Studies that reported aggressive behaviors in individuals with AD and MCI compared with healthy individuals or those with other dementia etiologies were identified. Risks of aggressive behaviors were assessed using random effects models to calculate pooled odds ratios (ORs). Publication bias was examined.

**Results:**

In total, 17 studies involving 6,399 individuals with AD and 2,582 with MCI were identified. Compared with healthy individuals, significantly increased risks of aggressive behaviors were found in AD (OR, 4.9, 95% CI, 1.8–13.2) but not in MCI (OR, 1.8, 95% CI, 0.7–4.3). When comparing AD with MCI, the risk in AD was higher (OR, 2.6, 95% CI, 1.7–4.0). We found no differences in risk of aggressive behaviors between AD and other dementia subtypes or between amnestic and nonamnestic MCI.

**Conclusion:**

Individuals with AD are at higher risk of manifesting aggressive behaviors than healthy individuals or those with MCI. Our findings not only underscore the necessity of treatment of aggressive behaviors in AD but also highlight the importance of preventing the transition from MCI to AD.

## Highlights

• This is the first quantitative synthesis estimating the risk of aggressive behaviors in dementia and mild cognitive impairment.
• Individuals with Alzheimer's disease have five times higher odds of aggression than healthy controls.
• We found no differences in risks of aggression between mild cognitive impairment and normal population controls and between dementias of differing aetiologies.

## INTRODUCTION

Evidence increasingly suggests elevated risk of exhibiting aggressive behaviors in Alzheimer disease (AD) and mild cognitive impairment (MCI). Such aggressive behaviors are among the most frequent and disruptive behavioral complications of cognitive decline contributing to increased cost of care, hospitalization, caregiver burden, and risk of premature institutionalization. Aggressive behaviors in these conditions are associated with medication use and physical restraint.^1^, ^2^, ^3^ Antipsychotic use is of limited effectiveness and is associated with potentially harmful side effects, such as increased risk of stroke and death.^4^ Physical restraint has been associated with a multitude of adverse psychological and physical effects.^5^, ^6^ Recent work has also suggested that caregiver distress is more closely connected to aggressive behaviors than key symptoms of AD and MCI.^7^ Furthermore, aggressive behaviors are major contributors to the financial burden of these conditions, especially as they frequently lead to premature institutionalization.^8^, ^9^, ^10^, ^11^ The precise magnitude of the risk of aggressive behaviors in AD and MCI remains unknown, as wide variations in estimates have been reported. For example, increased odds of aggressive behaviors have been reported to vary from 2 to 11 in AD and from 0.5 to 4 in MCI.^12^, ^13^, ^14^, ^15^ To our knowledge, a quantitative analysis of primary studies has not been conducted. In this article, we used a quantitative approach to robustly estimate the risk of aggressive behaviors in AD and MCI. This could potentially aid caregivers, clinicians, and policy makers in facilitating planning of both patient care and public health policy.

## METHODS

### Search Strategy

We followed Preferred Reporting Items for Systematic Reviews and Meta-Analyses guidelines with a protocol registered with PROSPERO (registration CRD42017080952). Meta-analysis Of Observational Studies in Epidemiology (MOOSE) guidelines were followed. Studies of the association of AD, MCI, and aggressive behaviors were sought by searches of six computer-based databases (Medline, Embase, PubMed, PsycINFO, CINAHL, and Web of Science). We used combinations of key words related to MCI (cognitive impair*, cognitive decline*, and cognitive fail*), AD (dement*, alzheimer*, frontotemporal), and aggressive behaviors (aggress*, assault*, viol*, offen*, antisocial, anti-social, dangerous*, crim*, unlawful*). These were supplemented with scanning of article reference lists and correspondence with authors. Case-control and cohort studies were included if they investigated the risk of aggressive behaviors in individuals with AD and MCI compared with individuals without these disorders. We included studies that reported aggressive behaviors using validated scales such as the Neuropsychiatric Inventory (NPI) and the Behavioral Pathology in Alzheimer's Disease Rating Scale. AD and MCI were diagnosed by validated instruments or standard clinical interview. Studies were excluded if they did not provide information that allowed for the calculation of odds ratios (ORs). In addition, studies of individuals who were inpatients or in other institutional settings were excluded to avoid probable biases associated with these samples.^16^

### Data Extraction

A standardized form was used to extract data from the included studies. For every eligible study, the following information was extracted: numbers of individuals with and without AD/MCI by aggressive behavior status, age, sex breakdown, geographic location, year of publication, diagnostic instrument, study setting, study design, and informant. The information was recorded and coded according to a fixed protocol. Data were extracted and cross-checked by two authors (RY and AT). Discrepancies were resolved by further review, discussion among RY and AT, and consultation with SF.

### Statistical Analysis

To synthesize the evidence from the literature, we conducted meta-analyses. Analyses were conducted in Stata 14 (StataCorp, College Station, TX). ORs with 95% confidence intervals (CIs) of the risk of aggressive behaviors in AD or MCI compared with healthy subjects were combined using meta-analysis. We also conducted comparisons between AD and MCI, AD subtypes, and MCI subtypes. The data were presented in forest plots. Random effects models, which incorporate an estimate of between-study heterogeneity into the calculation of the common effect, were used, as the heterogeneity between studies was high.^17^ Random effects estimates can give relatively similar weight to studies of different size.

Heterogeneity between studies was estimated using the *I*^2^ statistic, which describes the percentage of variation across studies because of heterogeneity rather than chance. *I*^2^does not inherently depend on the number of studies considered. For *I*^2^, the values of 25%, 50%, and 75% indicate low, moderate, and high levels of heterogeneity, respectively.^18^ Publication bias was tested by funnel plot asymmetry using the rank correlation method.^19^

## RESULTS

**FIGURE 1:**
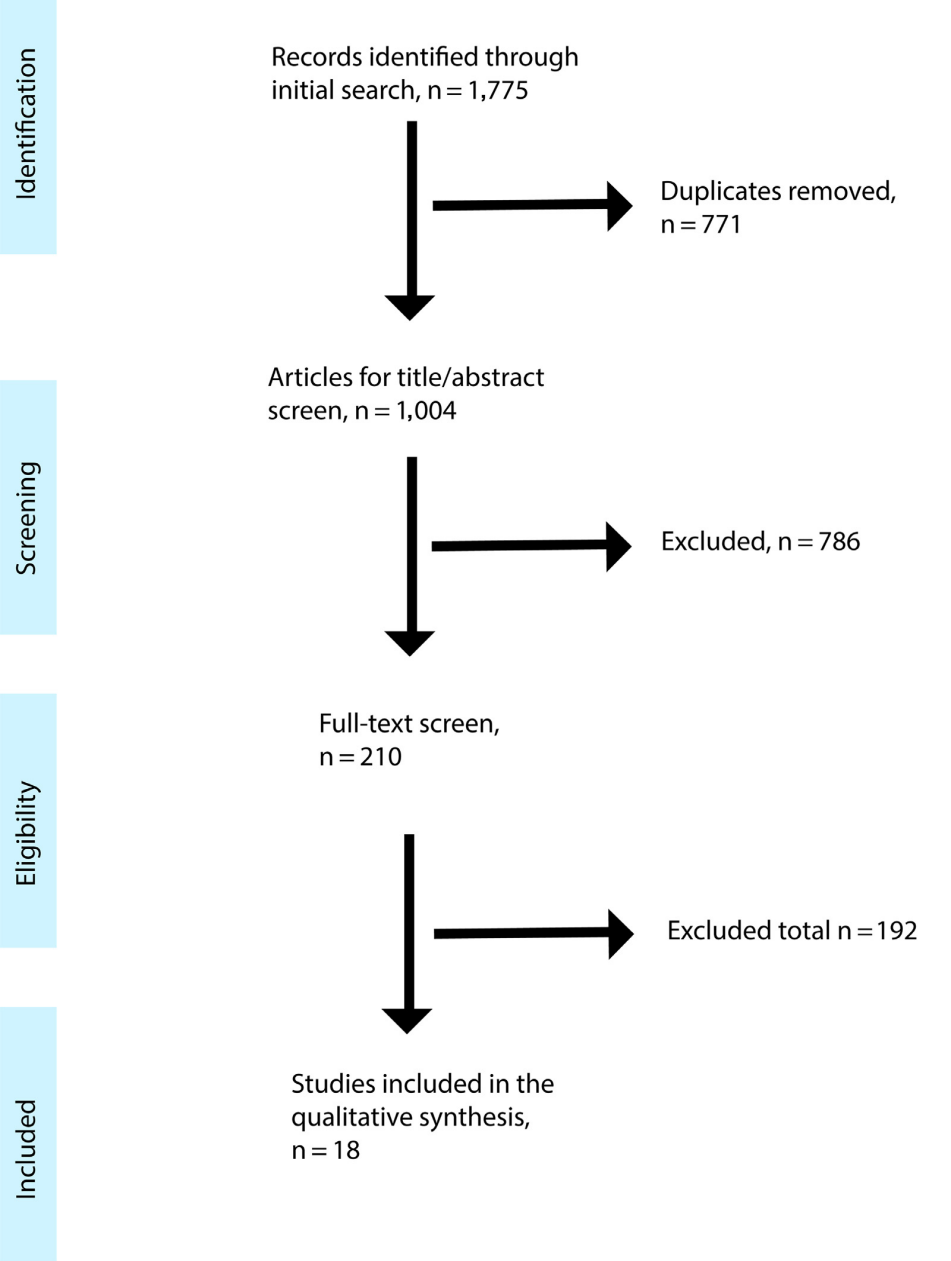
Flow chart of the systematic search strategy.

Figure 1 provides details of the study selection process. The final sample consisted of 17 studies, with 6,399 individuals with AD and 2,582 persons with MCI. A large proportion of studies were conducted in the United States (9 out of 17); the rest were from Belgium, Nigeria, the United Kingdom, Tanzania, Brazil, Japan, Taiwan, and South Korea. All studies were conducted after the year 1999. The most commonly used scale for the outcomes was the NPI (9 out of 17). Details of the included studies are summarized in Table 1.

**TABLE 1:** Characteristics of Included Studies on Risk of Aggression in Alzheimer Disease and Mild Cognitive Impairment

Study	Country	Comparisons	AD n	MCI n	Comparison Group (n)	Outcome	Design	Measurements	Informant	Age Range/Mean Age (Years)	Female %
Baiyewu 2012^12^	Nigeria	AD versus MCI versus HC	34	53	21	Agitation/aggression	Cross-sectional	NPI	Yes	AD: 83, MCI: 81, HC: 83	87
Lyketsos 2002^13^	United States	AD versus MCI versus HC	362	320	653	Arrest/crime for public order, driving or violent offense	Cross-sectional	NPI	Yes	70–80	32
Paddick 2015^14^	Tanzania	AD versus MCI versus HC	78	46	172	Agitation/aggression	Cross-sectional	NPI	Yes	AD: 85, MCI: 82, HC:^a^ 78	80
Tatsch 2006^15^	Brazil	AD versus MCI versus HC	60	25	78	Agitation/aggression	Cross-sectional	NPI	Yes	70–80	71
Gallagher 2011^20^	United Kingdom	AD versus MCI	69	92		Aggression	Longitudinal	BEHAVE-AD	Yes	AD: 75, MCI: 73	43
Mussele 2015^24^	Belgium	AD versus MCI	393	268		Aggression	Cross-sectional	CMAI & BEHAVE-AD	Yes	50–97	62
Liljegren 2015^21^	United States	AD versus MCI	545	243		Criminal behaviors	Retrospective	Electronic database	No	59–71	37
Lopez 2005^22^	United States	AD versus MCI	427	228		Aggression	Cross-sectional	Interview with psychiatrist	Yes	MCI: 70, AD: 73	60
Rockwood 2015^23^	United States	AD versus MCI	388	684		Aggression	Cross-sectional	Symptom guide based on NPI	Yes	72	58
Lee 2008^27^	South Korea	MCI subtypes	0	7	210	Agitation/aggression	Cross-sectional	NPI	Yes	70–75	65
Edwards 2009^25^	United States	MCI subtypes	50	56	328	Aggression	Cross-sectional	Clinical interview	Yes	n/a	53
Ellison 2008^26^	United States	MCI subtypes	16	4	18	Agitation/aggression	Cross-sectional	NPI	Yes	75	20
Ikeda 2004^29^	Japan	AD versus other dementia	21		60	Agitation/aggression	Cross-sectional	NPI	Yes	82	86
Lyketsos 1999^32^	United States	AD versus other dementia	296		99	Agitation/aggression	Cross-sectional	Patel and Hope definition	Yes	75	75
Chiu 2006^28^	Taiwan	AD versus other dementia	75		28	Aggression	Retrospective	BEHAVE-AD	Yes	72	78
Orengo 2008^30^	United States	AD versus other dementia	82		70	Aggression	Cross-sectional	Ryden Aggression Scale	Yes	n/a	1
Sadak 2013^31^	United States	AD versus other dementia	3338		239	Agitation/aggression	Cross-sectional	NPI	Yes	79	59

*Notes:* BEHAVE-AD: Behavioral Pathology in Alzheimer's Disease Rating Scale; CMAI: Cohen-Mansfield Agitation Inventory; HC: healthy individuals; n/a: not applicable; NPS: neuropsychiatric symptoms.

a Median (interquartile range).

Of the 17 studies included, four provided data on aggressive behaviors in AD and MCI, with healthy elderly as the comparison group.^12^, ^13^, ^14^, ^15^ Nine studies provided data for comparison between AD and MCI,^12^, ^13^, ^14^, ^15^,^20^, ^21^, ^22^, ^23^, ^24^ three for comparison between amnestic and nonamnestic MCI,^25^, ^26^, ^27^ and five comparing AD and other types of dementia.^13,^^28^, ^29^, ^30^, ^31^ Of the individuals with AD, 27.8% (n = 2,321) had aggressive behaviors compared with 7.4% (n = 306) of those with MCI and 5.8% (n = 54) of the healthy elderly. Aggressive behaviors included physical aggression, verbal outbursts, agitation, and crime. The overall prevalence of aggression in AD patients was 28% in population-based studies^12^, ^13^, ^14^, ^15^,^27^, ^29^ and 23% in samples from memory clinics.^20^, ^21^, ^22^,^24^, ^25^, [Bibr bib0026][Bibr bib0028][Bibr bib0030], 31, 32 The prevalence of aggression in MCI patients was 11% in population-based studies^12^, ^13^, ^14^, ^15^^,^^24^ and 12% in samples from memory clinics.^20^, ^21^, ^22^^,^^24^, ^25^, ^26^^,^^32^

**FIGURE 2:**
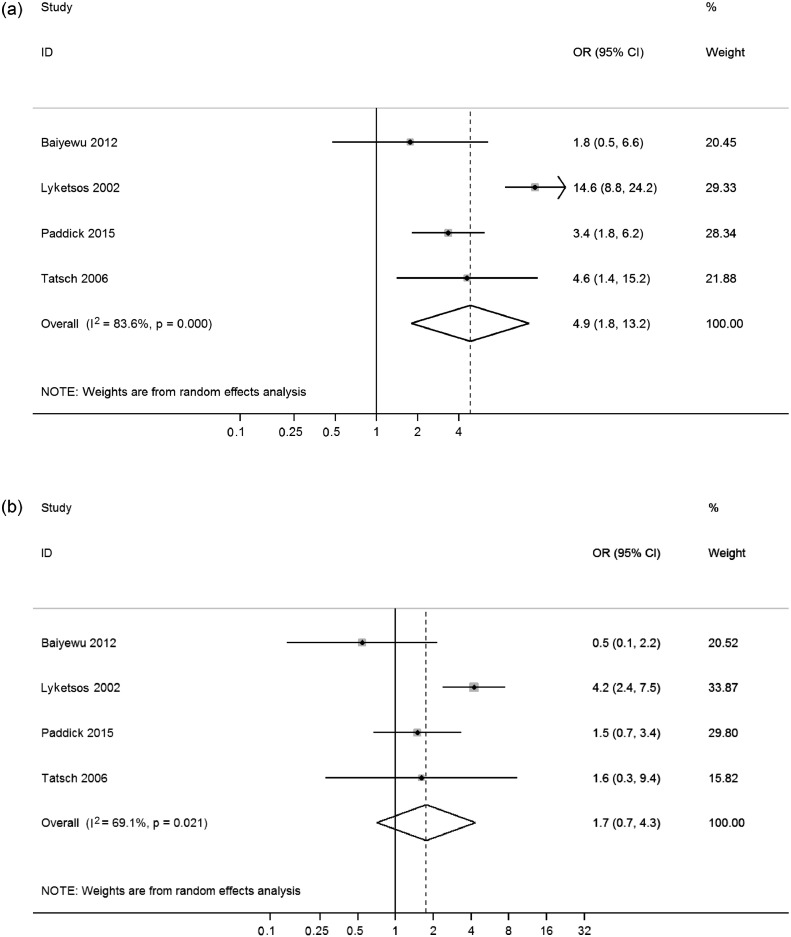
Risk of aggressive outcomes in Alzheimer disease (a) and mild cognitive impairment (b) compared with healthy individuals. ID: identification.

**FIGURE 3:**
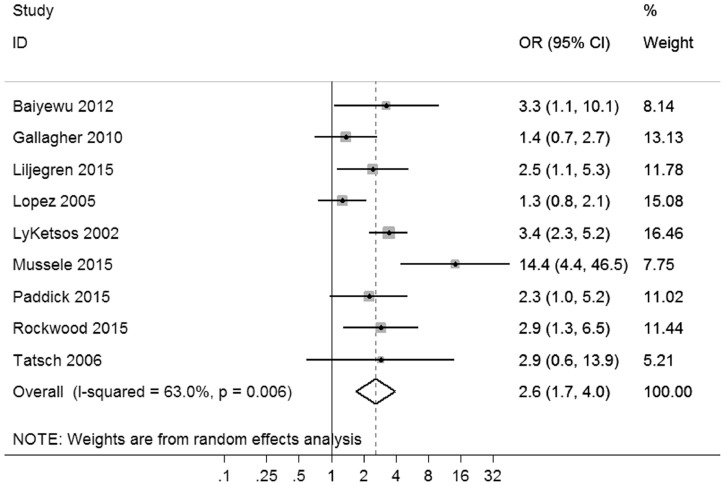
Risk of aggressive outcomes in Alzheimer disease compared with mild cognitive impairment. ID: identification.

**FIGURE 4:**
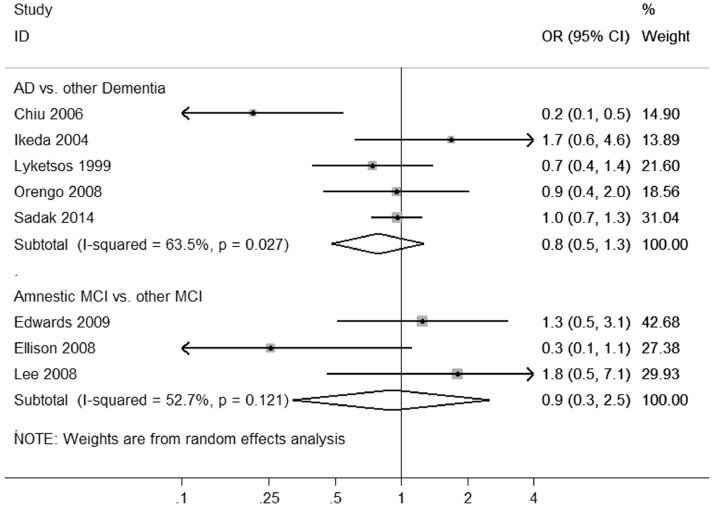
Comparisons of risk of aggressive outcomes among dementia subtypes and mild cognitive impairment subtypes. ID: identification.

Meta-analyses showed that AD was associated with increased odds of aggressive behaviors compared with healthy elderly (OR, 4.9, 95% CI, 1.8–13.2), with high heterogeneity across studies ($x_{3}^{2}$ = 18.3, p < 0.001, *I*^2^ = 84%) (Fig. 2a). In contrast, MCI was not significantly associated with aggressive behaviors. The overall random effects pooled OR was 1.8 (95% CI, 0.7–4.3), with moderate heterogeneity between studies ($x_{3}^{2}$ = 9.7, p = 0.02, *I*^2^ = 69%) (Fig. 2b). The risk of aggressive behaviors in AD was higher than in MCI (OR, 2.6, 95% CI, 1.7–4.0), with moderate heterogeneity ($x_{8}^{2}$ = 21.6, p = 0.01, *I*^2^ = 63%) (Fig. 3). Risks were similar for AD compared with other dementia subtypes, such as vascular and frontotemporal dementia (OR, 0.9, 95% CI, 0.3–2.5) (Fig. 4), and there were no significant differences in risk of aggressive behaviors when comparing amnestic and nonamnestic MCI ($x_{4}^{2}$ = 11.0, p = 0.03) (Fig. 4).

In the comparison between cases (AD and MCI) and healthy individuals, three studies provided data on the number of men and women in the AD group^12^, ^13^, ^14^ and four studies provided these data in the MCI group.^12^, ^13^, ^14^, ^15^ The proportion of men in each sample explained some of the differences between studies. We observed that studies with the lowest percentages of men (18% in the AD sample and 8% in the MCI sample) reported the lowest ORs in the comparisons between AD and healthy elderly (OR, 1.8, 95% CI, 0.5-6.6) and between MCI and healthy elderly (OR, 0.5, 95% CI, 0.1–2.2). In contrast, the studies with the highest percentages of men showed the highest risk. ORs were 14.6 (95% CI, 8.8-24.2) in the comparison between AD and healthy individuals and 4.2 (95% CI, 2.4-7.5) in the comparison between MCI and healthy individuals. Because of limited data, we were not able to conduct subgroup analyses to examine sex differences. However, we calculated percentages of men in the patient group and conducted metaregression testing to test whether this explained between-study variations in the odds of aggression. We found no association in the AD group (t = -0.09, p = 0.94, df = 2) and a nonsignificant association in the MCI group (t = 3.11, p = 0.09, df = 3).

In addition, we conducted subgroup analyses comparing the risk of aggression between AD and MCI by study setting. The pooled OR was 3.2 (95% CI, 2.2-4.5) in population-based studies and 2.5 (1.0-5.9) in samples from memory clinics. Because of limited data, we were not able to do subgroup analyses by study setting in other comparisons. In particular, all four studies that compared AD and MCI to healthy individuals were population-based.^12^, ^13^, ^14^, ^15^ Among the five studies comparing AD with other types of dementia, only one was population-based;^29^ the other four were from memory clinics. All three studies comparing amnestic MCI with nonamnestic MCI were from memory clinics.^25^, ^26^, ^27^

We found no evidence of publication bias in studies comparing AD and healthy individuals (t = -1.16, p = 0.37, df = 3), MCI and healthy individuals (t = -1.72, p = 0.23, df = 3), or AD and MCI (t = -0.31, p = 0.76, df = 8); those comparing MCI subtypes (t = -0.59, p = 0.66, df = 2); or those comparing AD and other dementias (t = -0.66, p = 0.56, df = 4).

## CONCLUSION

### Main Findings

In this systematic review and meta-analysis, we identified 17 studies involving AD and MCI—and a total of 8,981 cases—based in nine countries. Overall, we found 27.8% of individuals with AD and 7.4% with MCI exhibited aggressive behaviors. In those with AD, this equated to pooled increased odds of around five compared with healthy individuals. In those with MCI, there were slightly elevated odds of around two, but this was not statistically significant. There was moderate to high heterogeneity in risk estimates between studies, with the proportion of male participants providing one explanation. In addition, we found no clear differences in the risk of aggressive behaviors between AD and other forms of dementia or between amnestic and nonamnestic MCI.

### Implications

Our study shows that the risks of exhibiting aggressive behaviors in AD are significantly higher than in healthy individuals. The high absolute risk of aggression in AD and the negative impact of these behaviors on patients themselves, caregivers, and healthcare services underscore the importance of these findings and the need for proactive management of aggression. Furthermore, they emphasize the need for prevention strategies. A recent Lancet Commission on dementia prevention estimated the total adjusted population attributable risk fraction (the percentage reduction in new cases over a given time if nine identified risk factors are completely eliminated) at 35%,^33^ which is likely an underestimate, as homocysteine levels and alcohol intake, which are modifiable, were not included.^34^, ^35^

Despite the high prevalence and wide range of negative outcomes in AD, currently there is a lack of effective and safe treatment options for aggressive behaviors. The most recent systematic review of antipsychotic medications for behavioral and psychological symptoms (which included but was not limited to aggression) in dementia identified 12 meta-analyses reporting modest effect sizes.^36^ Efficacy may be higher in hospital inpatients or those with more severe symptoms.^36^ Although antipsychotics may be more effective than nonpharmacologic strategies, their harms to patients is likely to be higher.^37^ Of particular concern is the higher risk of cerebrovascular events and death.^4^ In clinical practice, aggressive behaviors manifest along a severity spectrum, from aggressive resistance (usually occurring in the context of intimate care)^38^ to very rare extreme events such as homicide.^39^ Given the risks, antipsychotics should be reserved for those with the most severe symptoms.^40^ Nonpharmacologic interventions, including environmental and behavioral modification, are safer alternatives for less severe symptoms and, possibly, MCI.^41^, ^42^

However, these interventions can be difficult to implement, especially in nursing home settings, where staff-to-resident ratios are frequently low.^43^ Recent research has shown preliminary evidence of the efficacy of electroconvulsive therapy in reducing aggressive behaviors in patients with dementia,^44^ although future studies are warranted to confirm these findings and the use of such therapy is likely to be limited to the most difficult cases.

To develop effective preventive and treatment strategies, a deeper understanding of risk factors and underlying mechanisms will be required. Studies have indicated that depression, chronic pain, loss of family contact, social deprivation, caregiver-patient relationships, and the nursing home environment might be related to aggression in AD.^45^, ^46^, ^47^ Future research is required to clarify the independence, strength, and interaction of these risk factors.

We found no significant difference in aggression between AD and other forms of dementia. This might be because of limited data that restricted group comparisons. Clinical experience suggests that there could be differences between, for instance, AD and frontotemporal dementia. It has been reported that the prevalence of criminal behaviors is 37% in frontotemporal dementia and 8% in AD.^21^ However, we found insufficient data to investigate these different dementia presentations using meta-analytic methods. Our analyses indicate that the risk of aggression is similar in both amnestic and nonamnestic MCI even though amnestic MCI predominantly affects short-term memory, whereas nonamnestic MCI is characterized by disturbances in attention/concentration, information processing, psychomotor speed, language, and executive function.^48^ There were insufficient studies examining MCI subgroups of different etiology (e.g., vascular versus nonvascular) and functional status (e.g., MCI-I versus MCI-II). Additional studies and individual patient meta-analyses may facilitate evidence synthesis.

### Sex and Other Factors

Among the studies providing data on the number of men and women,^12^, ^13^, ^14^, ^15^ we found that those with the highest proportion of men reported the highest prevalence of aggression. This is consistent with reports examining other disruptive behavioral problems, such as wandering, abusiveness, and social impropriety.^49^ Sex differences in behavioral problems associated with AD could affect treatment decisions. For instance, psychoactive medications are more likely to be used for the treatment of behavioral disturbances in men with AD than in women.^49^ Nevertheless, the mechanism behind the sex differences remains unknown, and future studies are needed to investigate this and potentially inform treatment strategies.

We found that the absolute prevalence of aggression in AD and MCI was similar in both population-based and memory clinic samples. This finding is not inconsistent with the possibility that cases referred to memory clinics are more likely to be deemed more cognitively impaired and behaviorally challenged^15^, ^50^ because of the way aggression is measured. In all of the population-based studies, aggression was measured with an NPI questionnaire that combined aggression and agitation, whereas aggression was measured in memory clinics with additional specific questionnaires.^21^, ^25^

In addition, we found that the absolute rate of aggression in AD was higher in memory clinic samples than population-based samples, although the rates of aggression in MCI were similar in these two settings. Furthermore, subgroup analyses by study setting demonstrated similar relative risk in the comparison between AD and MCI. Because of limited data, analyses were not conducted to explore other factors, such as diagnostic instrument, that might contribute to heterogeneity between studies.

### Limitations

A number of limitations should be noted. First, the majority of included studies used the Behavioral Pathology in Alzheimer's Disease Rating Scale, the Cohen-Mansfield Agitation Inventory, or the NPI to identify aggressive behaviors. These instruments capture information from only the preceding 2–4 weeks. Therefore, any calculations based on them are underestimates of the absolute risk of aggression throughout the course of a cognitive disorder, although any underestimation of relative risks will depend on whether the time at risk in the comparison group is similar. Second, we were unable to distinguish aggression from agitation in 9 out of 17 studies, as the recording instruments (such as the NPI) combined these two behaviors. Likewise, we could not separately examine risks of physical and verbal aggression. In addition, one included study suggested that AD was related to criminal behaviors, which is consistent with a previous study reporting dementia to be prevalent (7%) in older mentally disordered offenders.^52^ However, more studies examining the link between dementia and crime are necessary. As these outcomes are of varying severity and lead to different consequences, in terms of treatment, security management, caregiver training, and protection, it is important to know the risk of these behaviors separately. Third, risks were based on behaviors that were reported by an informant, and it is possible that some behaviors might not be witnessed or recalled. This suggests that our reported risks are likely to be underestimates. Fourth, caution should be taken in interpreting the estimates, as there was substantial heterogeneity between studies. Future studies are needed to confirm whether severity or etiology of cognitive impairment—or psychiatric comorbidity such as depressive or psychotic symptoms—could alter the risk of aggression in AD.^51^ Finally, aggression estimates from the included studies may not generalize to other populations. Some studies included individuals with low levels of education or literacy,^12^, ^27^^,^^28^ and one was conducted in mostly male veterans.^30^ In addition, two investigations examined patients seen at tertiary memory assessment centers and may not reflect those seen in other care settings.^21^^.^^25^

In summary, this meta-analysis reports a fivefold increase in the odds of aggressive behaviors in individuals with AD compared with healthy individuals. Our findings not only underscore the necessity of treatment and management of aggressive behaviors in AD but also highlight the importance of preventing the transition from MCI to AD. Further research is necessary to examine the role of other risk factors for aggression, including psychiatric comorbidity and environmental characteristics, and whether more accurate risk prediction can improve outcomes.
